# Vitamin D status among patients visiting a tertiary care center in Riyadh, Saudi Arabia: a retrospective review of 3475 cases

**DOI:** 10.1186/1471-2458-14-159

**Published:** 2014-02-13

**Authors:** Hanan Alfawaz, Hani Tamim, Shmeylan Alharbi, Saleh Aljaser, Waleed Tamimi

**Affiliations:** 1 Department of Food Science and Nutrition, College of Food Science and Agriculture King Saud University, P. O. Box 22452, Riyadh 11495, Saudi Arabia; 2 Department, College of Science, Prince Mutaib Chair for Biomarkers of Osteoporosis, Biochemistry, King Saud University, Riyadh, Saudi Arabia; 3 Department of Internal Medicine, Clinical Research Institute, American University of Beirut Medical Center, Beirut, Lebanon; 4 Pharmaceutical Care Department, College of Pharmacy, King Saud bin Abdulaziz University for Health Sciences, Riyadh, Saudi Arabia; 5 Diabetes Care Program, King Abdulaziz Medical City, Riyadh National Guard, Riyadh, Saudi Arabia; 6 College of Medicine, King Saud Bin Abdulaziz University for Health Sciences, Riyadh, Saudi Arabia; 7 Department of Pathology & Lab Medicine, College of Medicine, King Saud Bin Abdulaziz University for Health Sciences, Riyadh, Saudi Arabia

**Keywords:** Vitamin D, Vitamin D deficiency, Saudi

## Abstract

**Background:**

Vitamin D deficiency has been implicated in several chronic, non-communicable diseases independent of its conventional role in bone and calcium homeostasis. In this retrospective study, we determined the prevalence of vitamin D deficiency and its association to several cardiometabolic indices among patients visiting King Abdulaziz Medical City (KAMC), a tertiary hospital in Riyadh, Saudi Arabia.

**Methods:**

A total of 3475 charts of out-patient subjects who visited KAMC from September 2009 until December 2010 were reviewed and included. Variables of interest included measurements of vitamin D status, glycemic and renal profile, as well as trace elements (calcium and phosphorous).

**Results:**

The over-all prevalence of vitamin D deficiency in the cohort studied was 78.1% in females and 72.4% in males. 25(OH) vitamin D was significantly associated with increasing age and weight (*p*-values < 0.0001 and 0.005, respectively). It was also positively associated with albumin, calcium and phosphorous (*p*-values < 0.0001, < 0.0001 and 0.0007, respectively) and negatively associated with alkaline phosphatase as well as circulating levels of PTH (*p*-values 0.0002 and 0.0007, respectively).

**Conclusion:**

In conclusion, vitamin D deficiency is overwhelmingly common among patients seen at KAMC regardless of the medical condition, and it is significantly associated with increasing age, weight and markers of calcium homeostasis. Findings of the present study further stress the spotlight on vitamin D deficiency epidemic in the country and region in general.

## Background

Recent epidemiologic studies have found out an unpredictably high prevalence of vitamin D deficiency in apparently healthy adults living in different countries, which could be a major health problem in the future [1,2]. Adequate vitamin D status has important clinical advantages in decreasing risk of many diseases such as cancer [3-6], diabetes mellitus, cardiovascular [7] and autoimmune diseases [8]. Evidences from clinical and epidemiological studies support a possible relationship between low vitamin D status and chronic disease progression such as obesity, hypertension and diabetes mellitus [9-11]. Studies in Saudi Arabia, the United Arab Emirates, Australia, Turkey, India, and Lebanon, reported that 30 to 50% of children and adults had 25-hydroxyvitamin D levels under 20 ng/ml [12-15]. Saudi Arabia belongs to one of the sunniest regions in the world, and while the Saudi population should have adequate sun exposure, vitamin D deficiency remains prevalent in the country [16]. Various reasons include protection from strong heat during daytime, genetic and diet. Vitamin D deficiency was found to be very common among Saudi males and females [16-20]. Ardawi *et al*. found that vitamin D deficiency was common among older and obese Saudi men [21]. Findings from Al-Daghri *et al*. indicated severe hypovitaminosis D as more common among non-diabetic than diabetic Saudis [22]. Several studies have also reported conflicting findings on the relationship between vitamin D status and obesity. Results of Al-Elq *et al.* study found an inverse relationship between vitamin D and BMI in Saudi males but not in females which appears that obesity is protective against vitamin D deficiency [23]. While negative association was found in many studies [24-28], some observed no relationship [29,30]. The mechanism behind such an association is that elevated concentrations of 1-25-vit D stimulate lipogenesis and inhibit lipolysis in cultured human adipocytes, leading to accumulation of fat [31]. Additionally, 1, 25-vitamin D inhibits the expression of adipocyte uncoupling protein 2 (UCP2), which would cause a reduction in the adipocyte’s metabolic efficiency [32]. Cumming *et al.* found that vitamin D and calcium were more effective in reducing systolic blood pressure than calcium alone [33]. Furthermore, many studies reported a positive association between 1, 25(OH) 2D and vitamin D inadequacy and hypertension [34-36]. Some studies nevertheless reported conflicting results on the link between vitamin D intake and blood pressure [37,38]. Low serum vitamin D levels elevate the risk for early-stage diabetes (Pre-DM), hypertension (Pre-HTN) [37] and DM [39-41]. On the other hand, many studies reported no association between vitamin D deficiency and type 2 diabetes mellitus [42,43]. In the present study, we examined the relationship between serum levels of 25-hydroxyvitamin D (25[OH] D), Parathyroid Hormone (PTH), obesity and selected cardiovascular disease risk factors in Saudi subjects.

## Methods

In this single-center retrospective study done in the outpatient department of King Abdulaziz Medical City, Riyadh, Saudi Arabia, a total of 3475 subjects’ charts were reviewed from September 2009 until December 2010. There were 2719 (78%) females and 756 (22%) males. Vitamin D (25[OH] D) was measured using High Performance Liquid Chromatography (HPLC). Data was collected from the laboratory master database of the Clinical Biochemistry section, Department of Pathology and Laboratory Medicine, in KAMC. In addition to vitamin D2, other laboratory tests such as fasting blood sugar (FBG), HbA1C, and PTH were also noted.

The corresponding medical record number of those patients were utilized to obtain the following information from Quadramed and/or medical files: Height, weight, blood pressure, HbA1c, albumin, creatinine, BUN, alkaline phosphatase, calcium, phosphorous and parathormone. All clinical parameters were measured at the same time or close to the date of vitamin D measurement. The study has been approved by the [Institutional Review Board (IRB)] Clinical Research Ethics Committee in KAMC.

### Data analysis

Data was analyzed using the Statistical Package for the Social Sciences (SPSS version 16.0, Chicago, IL, USA). Frequencies were expressed in percentages (%) and continuous variables were presented as mean ± standard deviation. Variables that were not normally distributed (alkaline phosphatase, BUN, creatinine, 25(OH) vitamin D, and PTH) were transformed and normalized prior to parametric analysis (Pearson bivariate correlation). Student independent T-test was done to compare means of normally distributed variables and Mann–Whitney U-test for variables that are non-Gaussian. Chi-Square test was used to compare frequencies. Significance was set at *p* < 0.05.

## Results

**Figure 1:**
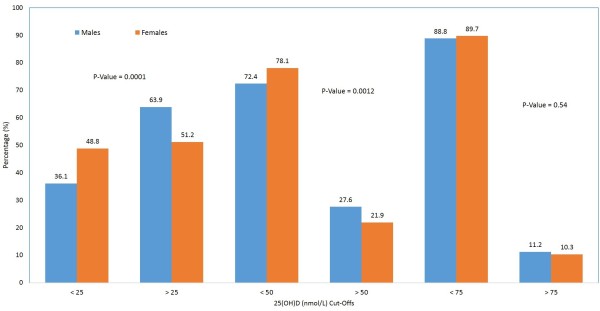
Prevalence of vitamin D deficiency in males and females according to vitamin D cut-offs.

Figure 1 shows the differences in the prevalence vitamin D deficiency according to severity across genders using different cut-off values. Females had a significantly higher prevalence of 25(OH)D < 25 nmol/L than males (48.8% versus 36.1%; *p*-value 0.0001) as well as a higher prevalence of 25(OH) vitamin D < 50 nmol/L (78.1% versus 72.4%; *p*-value 0.0012). Using the same cut-off, it can be observed that the over-all prevalence of vitamin D deficiency in the cohort studied was 78.1% in females and 72.4% in males (Figure 1).

Table 1 describes the general characteristics of subjects and the associations of 25(OH) vitamin D to the different parameters measured. 25(OH) vitamin D was significantly associated with increasing age and weight (*p*-values < 0.0001 and 0.005, respectively). Furthermore, 25(OH) vitamin D was modestly, but significantly associated with increasing systolic blood pressure (*p* = 0.03). Among the biochemical parameters, 25(OH) vitamin D was positively associated with albumin, calcium and phosphorous (*p*-values < 0.0001, < 0.0001 and 0.0007, respectively) and negatively associated with alkaline phosphatase as well as circulating levels of PTH (*p*-values 0.0002 and 0.0007, respectively). It is worthy to note that the prevalence of obesity in the cohort studied is 21.9%, while the prevalence of morbid obesity was 44.2% (not shown in table).

**Table 1:** General characteristic of subjects

**Parameters**	**Mean ± SD**	**R**^**2**^	**P-value**
N = 3475 (Males = 756; Females = 2719)			
Age (years)	46.9 ± 16.3	0.15	< 0.0001
Weight (kg)	75.2 ± 18.2	−0.06	0.005
Height (cm)	147.0 ± 18.0	0.01	0.64
BMI (kg/ m^2^)	36.4 ± 13.8	−0.03	0.17
Systolic blood pressure (mmHg)	122.0 ± 19.0	0.05	0.03
Diastolic blood pressure (mmHg)	71.0 ± 11.0	0.04	0.09
Glucose (mmol/L)	6.4 ± 3.0	0.02	0.24
HbA1c (%)	7.0 ± 1.9	0.01	0.70
Albumin (g/L)	46.0 ± 4.9	0.11	<0.0001
Alkaline phosphatase (U/L)#	97.1 ± 82.7	−0.09	0.0002
BUN (mmol/L)	5.1 ± 3.4	0.03	0.12
Calcium (mmol/L)	2.34 ± 0.15	0.14	<0.0001
Creatinine (umol/L)#	79.4 ± 70.2	−0.01	0.48
25OH Vitamin D (nmol/L)#	35.5 ± 30.6	1.00	--
Phosphorus (mmol/L)	1.14 ± 0.23	0.09	0.0007
PTH (mmol/L)#	34.2 ± 58.3	−0.10	0.0007

**Note**: Data presented as mean ± standard deviation; R^2^ is the correlation coefficients compared to the concentration of 25OH Vitamin D; #denotes non-Gaussian variable.

Table 2 shows the comparison of the different variables using different cut-offs for 25(OH) vitamin D. Across all groups, subjects categorized to be in the upper half (≥ 25, ≥ 50 and ≥ 75 nmol/L) were significantly older (*p*-values < 0.0001, < 0.0001 and 0.0003, respectively) and had significantly higher levels of serum albumin (*p*-values 0.0002, < 0.0001 and 0.0021, respectively) and calcium (*p*-values < 0.0001, < 0.0001 and 0.0029, respectively) than those in the lower half. In the first grouping (< 25 and ≥ 25 nmol/L), subjects whose 25(OH) vitamin D levels were ≥ 25 nmol/L had significantly higher systolic and diastolic blood pressure as well as BMI (*p*-values < 0.0001, 0.0017 and < 0.0001, respectively) than subjects with 25(OH) vitamin D < 25 nmol/L). Among the biochemical parameters measured, those in the upper half (≥ 25 nmol/L) had a significantly higher blood fasting glucose, BUN and phosphorous (p-values < 0.0001, 0.0002 and < 0.0001, respectively) as well as a significantly lower alkaline phosphatase (*p*-value = 0.0001) than the lower half (< 25 nmol/L). In the second grouping, serum alkaline phosphatase was also observed to be significantly lower in the upper half (≥ 50 nmol/L) as compared to the lower half (<50 nmol/L) (*p*-value = 0.0082). Both the upper half of the 2nd and 3rd group (≥ 50 and ≥ 75 nmol/L) had significantly lower PTH levels (*p*-values = 0.034 and 0.039, respectively) than their corresponding lower halves (< 50 and < 75 nmol/L). The rest of the comparisons done for other variables not mentioned in all groups were non-significant. Worthy of mention however is the mean HBA1c among subjects whose 25(OH) vitamin D is > 75 nmol which, while not significant, is considered the lowest as compared to the rest of the groups.

**Table 2:** Comparison of variables using different vitamin d cut-off values (t-test)

**Parameters**	***25 (OH) vitamin D cut-offs (nmol/L)***					
	**< 25**	**≥ 25**	**< 50**	**≥ 50**	**< 75**	**≥ 75**
N	1601	1874	2672	803	3110	365
Age (years)	43.5 ± 16.3	49.9 ± 15.8**	45.7 ± 16.3	51.0 ± 15.8**	46.6 ± 16.3	49.8 ± 15.8**
BMI (kg/ m^2^)	35.1 ± 12.6	37.9 ± 14.8**	37.1 ± 14.9	33.8 ± 11.5**	37.0 ± 14.0	30.3 ± 7.7**
Systolic blood pressure (mmHg)	120.0 ± 19.0	124.0 ± 19.0**	122.0 ± 19.0	123.0 ± 17.0	122.0 ± 19.0	121.0 ± 16.0
Diastolic blood pressure (mmHg)	70.0 ± 11.0	72.0 ± 11.0**	71.0 ± 11.0	71.0 ± 11.0	71.0 ± 11.0	70.0 ± 10.0
Glucose (mmol/L)	6.2 ± 2.6	6.5 ± 3.4**	6.4 ± 2.9	6.4 ± 3.4	6.4 ± 3.0	6.6 ± 2.8
HbA1c (%)	6.9 ± 1.8	7.1 ± 1.9	7.1 ± 1.9	6.9 ± 1.8	7.0 ± 1.9	6.8 ± 1.9
Albumin (g/L)	45.6 ± 5.1	46.3 ± 4.5**	45.7 ± 5.0	46.9 ± 3.9**	45.9 ± 4.9	46.9 ± 4.0**
Alkaline phosphatase (U/L)	106.0 ± 103.0	90.0 ± 60.0**	100.0 ± 88.0	88.0 ± 69.0**	98.0 ± 85.0	91.0 ± 58.0
BUN (mmol/L)	4.9 ± 3.5	5.3 ± 3.3**	5.0 ± 3.5	5.2 ± 2.8	5.1 ± 3.5	5.0 ± 2.2
Calcium (mmol/L)	2.3 ± 0.2	2.4 ± 0.2**	2.3 ± 0.2	2.4 ± 0.1**	2.33 ± 0.15	2.37 ± 0.15**
Creatinine (umol/L)	77.0 ± 65.0	82.0 ± 74.0	80.0 ± 75.0	77.0 ± 53.0	80.0 ± 74.0	73.0 ± 21.0
Phosphorus (mmol/L)	1.11 ± 0.24	1.16 ± 0.22**	1.13 ± 0.24	1.15 ± 0.21	1.13 ± 0.23	1.15 ± 0.22
PTH (pmol/L)	38.0 ± 67.0	31.0 ± 51.0	36.0 ± 62.0	28.0 ± 48.0**	36.0 ± 61.0	25.0 ± 35.0*

**Note:** Data presented as mean ± standard deviation; *denotes significance at < 0.05 level; **denotes significance at < 0.01 level.

## Discussion

The major finding in the present one-year retrospective study is the overwhelming prevalence of vitamin D deficiency among Saudi patients seen at the outpatient clinics of KAMC. This confirms, and adds to the increasingly accumulating evidence that vitamin D deficiency in Saudi Arabia, specifically in urban areas such as the capital Riyadh, is alarmingly high [12-22]. Furthermore, the results of local epidemiologic findings on vitamin D deficiency, including the present study, are undeniably consistent, regardless of the methods used to quantify 25(OH)D, strengthening the premise that vitamin D deficiency is an epidemic in Saudi Arabia. What makes the present study unique is the arguably larger sample size as compared to previous studies, and that the type of cohort used can be considered representative of the general population, since patients referred to KAMC are not limited to the capital Riyadh, and that inclusion of cases was not stringent, making the selection of cases devoid of bias.

Among the associations of 25(OH) vitamin D and cardiometabolic variables elicited, it is worthy to emphasize that vitamin D deficiency is less common among the elderly in the Saudi population. Looking back at the mean glucose and HBA1c levels of the cohort, it is apparent that majority of the subjects included had diabetes mellitus type 2 (DMT2), and age-related disease. Subjects who harbor this disease have been observed to have higher levels of 25(OH) D than their non-diabetic counterparts [22-45]. Part of the explanation lies in the almost mandatory multivitamin supplementation (multivitamins contain 400 IU vitamin D on average) given to Saudi elderly patients and those with DMT2 as well as other anti-DM medications that have been observed to augment circulating levels of 25(OH) vitamin D [45,46]. Another obvious risk factor from the present cohort is obesity. The mean BMI for the entire cohort fell within this category (36.4 ± 13.8). Obesity is a well-known cardiovascular risk factor associated with vitamin D deficiency, more so for the Arab population where vitamin D correction has modest, if not negligible effect on BMI [47-49].

With regards to other biochemical parameters measured, the association of 25(OH) vitamin D to albumin is expected in the study. It has been established that majority of circulating vitamin D is bound both to vitamin D binding protein and albumin [50,51]. The same expected significant association is true for vitamin D and calcium, in which the former is directly involved in calcium homeostasis. Lastly, the lack of significant difference in PTH levels of those below and above 25 nmol/L is consistent with the findings of Al-Saleh *et al.*, where PTH levels remain normal despite having low to very low 25(OH) vitamin D levels, a unique feature among the Arabian cohort [52]. Nevertheless, the present study showed a significant and inverse association between PTH and vitamin D status, but differences in levels were only prominent if < 50 or < 75 nmol/L was used. Longitudinal studies are needed to confirm at what level of vitamin D status correction in the Arab cohort is needed to elicit a PTH response.

The study acknowledges several limitations. The retrospective and cross-sectional nature of the study as well as the population selected from the general outpatient clinics limit the findings to the information available at the database. The study was not able to consider confounding variables and other risk factors that can be used to adjust analysis such as presence of DMT2, skin color, sun exposure information and season where vitamin D was measured, the latter 2 factors being considered as very important predictors for vitamin D status in this geographical region [53]. Nevertheless, the study has several strengths and that includes the large sample size and the inclusion of all adult subjects whose vitamin D status was measured at a given time frame in one institution, which removed selection bias and increased the generalizability of present findings.

## Conclusion

In conclusion, vitamin D deficiency is overwhelmingly common among patients seen at KAMC regardless of the medical condition, further stressing the spotlight on this epidemic in the country and region in general. Aggressive measures should not be limited on the diagnosis, and multi-institutional involvement that includes policy makers, government and private companies should carry out public health campaigns to increase awareness and limit the spread of vitamin D deficiency engulfing the nation.

## Competing interests

The authors declare that they have no competing interests.

## Authors’ contributions

HF designed study, collected and revised data, drafted the initial and the final version of the manuscript. HT carried out data analysis and interpretation. SJ, SH and WT revised data and manuscript. All authors provided intellectual contributions to the manuscript and has read and approved the final version.

## Pre-publication history

The pre-publication history for this paper can be accessed here:

http://www.biomedcentral.com/1471-2458/14/159/prepub

## References

[B1] Rucker D Allan JA Fick GH Hanley DA Vitamin D insufficiency in a population of healthy western Canadians CMAJ 2002 166 1517 1524 12074117 PMC113796

[B2] Hasehmipour S Larijani B Adibi H Javadi E Sedaghat M Pajouhi M Soltani A Shafaei AR Hamidi Z Fard AR Hossein-Nezhad A Booya F Vitamin D deficiency and causative factors in the population of Tehran BMC Public Health 2004 4 38 10.1186/1471-2458-4-38 15327695 PMC517720

[B3] Garland CF Gorham ED Mohr SB Grant WB Giovanucci EL Lipkin M Newmark H Holick MF Garland FC Vitamin D and prevention of breast cancer: pooled analysis J Steroid Biochem Mol Biol 2007 103 708 711 10.1016/j.jsbmb.2006.12.007 17368188

[B4] Grant WB Epidemiology of disease risks in relation to vitamin D insufficiency Prog Biophys Mol Biol 2006 92 65 79 10.1016/j.pbiomolbio.2006.02.013 16546242

[B5] Grant WB How strong is the evidence that solar ultraviolet B and vitamin D reduce the risk of cancer? An examination using Hill’s criteria for causality Dermato-Endocrinology 2009 1 17 24 10.4161/derm.1.1.7388 20046584 PMC2715209

[B6] Grant WB A critical review of vitamin D and cancer: a report of the IARC working group on vitamin D Dermato-Endocrinology 2009 1 25 33 10.4161/derm.1.1.7729 20046585 PMC2715207

[B7] Wang TJ Pencina MJ Booth SL Jacques PF Ingelsson E Lanier K Benjamin EJ D’Agostino RB Wolf M Vasan RS Vitamin D deficiency and risk of cardiovascular disease Circulation 2008 117 503 511 10.1161/CIRCULATIONAHA.107.706127 18180395 PMC2726624

[B8] Munger KL Levin LI Hollis BW Howard NS Ascherio A Serum 25-hydroxyvitamin D levels and risk of multiple sclerosis JAMA 2006 296 2832 2838 10.1001/jama.296.23.2832 17179460

[B9] Lind L Hanni A Lithell H Hvarfner A Sorensen OH Ljunghall S Vitamin D is related to blood pressure and other cardiovascular risk factors in middle-aged men Am J Hypertens 1995 8 894 901 10.1016/0895-7061(95)00154-H 8541004

[B10] Hypponen E Laara E Reunanen A Jarvelin MR Virtanen SM Intake of vitamin D and risk of type 1 diabetes: a birth-cohort study Lancet 2001 358 1500 1503 10.1016/S0140-6736(01)06580-1 11705562

[B11] Chiu KC Chu A Go VL Saad MF Hypovitaminosis D is associated with insulin resistance and cell dysfunction Am J Clin Nutr 2004 79 820 825 15113720 10.1093/ajcn/79.5.820

[B12] Sedrani SH Low 25-hydroxyvitamin D and normal serum calcium concentrations in Saudi Arabia: Riyadh region Ann Nutr Metab 1984 28 181 185 10.1159/000176801 6610383

[B13] Marwaha RK Tandon N Reddy DR Aggarwal R Singh R Sawhney RC Saluja B Ganie MA Singh S Vitamin D and bone mineral density status of healthy schoolchildren in northern India Am J Clin Nutr 2005 82 477 482 16087996 10.1093/ajcn.82.2.477

[B14] El-Hajj Fuleihan G Nabulsi M Choucair M Salamoun M Hajj Shahine C Kizirian A Tannous R Hypovitaminosis D in healthy schoolchildren Pediatrics 2001 107 E53 10.1542/peds.107.4.e53 11335774

[B15] McGrath JJ Kimlin MG Saha S Eyles DW Parisi AV Vitamin D insufficiency in southeast Queensland Med J Aust 2001 174 150 151 11247622 10.5694/j.1326-5377.2001.tb143195.x

[B16] Sedrani SH Elidrissy AW Arabi KM Sunlight and vitamin D status in normal Saudi subjects Am J Clin Nutr 1983 38 129 132 6602540 10.1093/ajcn/38.1.129

[B17] Al-Turki H Sadat-Ali M Al-Elq A Al-Mulhim F 25-Hydroxyvitamin D levels among healthy Saudi Arabian women Saudi Med J 2008 29 1765 1768 19082230

[B18] Sadat-Ali M AlElq A Al-Turki H Al-Mulhim F Al-Ali A Vitamin D levels in healthy men in eastern Saudi Arabia Ann Saudi Med 2009 29 378 382 10.4103/0256-4947.55168 19700896 PMC3290044

[B19] Elsammak MY Al-Wosaibi AA Al-Howeish A Alsaeed J Vitamin d deficiency in Saudi Arabs Horm Metab Res 2010 42 364 368 10.1055/s-0030-1248296 20213587

[B20] Ardawi M Sibiany A Bakhsh T Qari M Maimani A High prevalence of vitamin D deficiency among healthy Saudi Arabian men: relationship to bone mineral density, parathyroid hormone, bone turnover markers, and lifestyle factors Osteoporos Int 2012 23 675 686 10.1007/s00198-011-1606-1 21625888

[B21] Ardawi M Qari M Maimani A Raddadi R Vitamin D status in relation to obesity, bone mineral density, bone turnover markers and vitamin D receptor genotypes in healthy Saudi pre- and postmenopausal women Osteoporos Int 2011 22 463 475 10.1007/s00198-010-1249-7 20431993

[B22] Al-Daghri NM Al-Attas OS Al-Okail MS Alkharfy KM Al-Yousef MA Nadhrah HM Sabico SB Chrousos GP Severe hypovitaminosis D is widespread and more common in non-diabetics than diabetics in Saudi adults Saudi Med J 2010 31 775 780 20635011

[B23] Al-Elq A Sadat-Ali M Al-Turki H Al-Mulhim F Al-Ali A Is there a relationship between body mass index and serum vitamin D levels? Saudi Med J 2009 301 542 546 19936417

[B24] Arunabh S Pollack S Yeh J Aloia JF Body fat content and 25-hydroxyvitamin D levels in healthy women J Clin Endocrinol Metab 2003 88 157 161 10.1210/jc.2002-020978 12519845

[B25] Parikh SJ Edelman M Uwaifo GI Freedman RJ Semega-Janneh M Reynolds J Yanovski JA The relationship between obesity and serum 1, 25-dihydroxy vitamin D concentrations in healthy adults J Clin Endocrinol Metab 2004 89 1196 1199 10.1210/jc.2003-031398 15001609

[B26] McGill A Stewart J Lithander F Strik S Poppitt S Relationships of low serum vitamin D3 with anthropometry and markers of the metabolic syndrome and diabetes in overweight and obesity Nutr J 2008 7 1 5 10.1186/1475-2891-7-1 18226257 PMC2265738

[B27] Konradsen S Jorde R Ag H Lindberg F Hexeberg S Serum 1,25-dihydroxy vitamin D is inversely associated with body mass index Eur J Nutr 2008 47 87 91 10.1007/s00394-008-0700-4 18320256

[B28] Macdonald H Mavroeidi A Barr R Black A Fraser W Reid D Vitamin D status in postmenopausal women living at higher latitudes in the UK in relation to bone health, overweight, sunlight exposure and dietary vitamin D Bone 2008 42 996 1003 10.1016/j.bone.2008.01.011 18329355

[B29] Epstein S Bell NH Shary J Shaw S Greene A Oexmann MJ Evidence that obesity does not in-fluence the vitamin D-endocrine system in blacks J Bone Miner Res 1986 1 181 184 3503535 10.1002/jbmr.5650010203

[B30] Nesby-O’Dell S Scanlon KS Cogswell ME Gillespie C Hollis BW Looker AC Allen C Doughertly C Gunter EW Bowman BA Hypovitaminosis D prevalence and determinants among African American and white women of reproductive age: third National Health and Nutrition Examination Survey, 1988–1994 Am J Clin Nutr 2002 76 187 12081833 10.1093/ajcn/76.1.187

[B31] Shi H Norman AW Okamura WH Anintida S Zemel MB 1α,25-dihydroxyvitamin D3 inhibits uncoupling protein 2 expression in human adipocytes FASEB J 2002 16 1808 1810 12223452 10.1096/fj.02-0255fje

[B32] Shi H Norman W Okamura WH Sen A Zemel MB 1α,25-dihydroxyvitamin D3 modulates human adipocyte metabolism via nongenomic action FASEB J 2001 15 2751 2753 11606486 10.1096/fj.01-0584fje

[B33] Cumming RG Cummings SR Nevitt MC Scott J Ensrud KE Vogt TM Fox K Calcium intake and fracture risk: results from the study of osteoporotic fractures Am J Epidemiol 1997 145 926 934 10.1093/oxfordjournals.aje.a009052 9149664

[B34] Sowers MF Wallace RB Hollis BW Lemke JH Relationship between 1, 25- dihydroxyvitamin D3 and blood pressure in a geographically defined population Am J Clin Nutr 1988 48 1053 1056 3421200 10.1093/ajcn/48.4.1053

[B35] Krause R Buhring M Hopfenmuller W Holick MF Sharma AM Ultraviolet B and blood pressure Lancet 1998 352 709 710 9728997 10.1016/S0140-6736(05)60827-6

[B36] Pfeifer M Begerow B Minne HW Nachtigall D Hansen C Effects of a short-term vitamin D(3) and calcium supplementation on blood pressure and parathyroid hormone levels in elderly women J Clin Endocrinol Metab 2001 86 1633 1637 11297596 10.1210/jcem.86.4.7393

[B37] Jorde R Bonaa K Calcium from dairy products, vitamin D intake, and blood pressure: the Tromso study Am J Clin Nutr 2000 71 1530 1535 10837295 10.1093/ajcn/71.6.1530

[B38] Forman JP Bischoff-Ferrari HA Willett WC Stampfer MJ Curhan GC Vitamin D intake and risk of incident hypertension results from three large prospective cohort studies Hypertension 2005 46 676 682 10.1161/01.HYP.0000182662.82666.37 16144983

[B39] Scragg R Sowers M Bell C Serum 25-hydroxyvitamin D, diabetes, and ethnicity in the Third National Health and Nutrition Examination Survey Diabetes Care 2004 27 2813 2818 10.2337/diacare.27.12.2813 15562190

[B40] Ford ES Ajani UA McGuire LC Liu S Concentrations of serum vitamin D and the metabolic syndrome among U.S. adults Diabetes Care 2005 28 1228 1230 10.2337/diacare.28.5.1228 15855599

[B41] Need AG O’Loughlin PD Horowitz M Nordin BE Relationship between fasting serum glucose, age, body mass index and serum 25 hydroxyvitamin D in postmenopausal women Clin Endocrinol (Oxf) 2005 62 738 741 10.1111/j.1365-2265.2005.02288.x 15943837

[B42] Pittas G Lau J Hu F Dawson-Hughes B The role of vitamin D and calcium in type 2 diabetes. A systematic review and meta-analysis J Clin Endocrinol Metab 2007 92 2017 2029 10.1210/jc.2007-0298 17389701 PMC2085234

[B43] Hidayat R Setiati S Soewondo P The association between vitamin D deficiency and type 2 diabetes mellitus in elderly patients Acta Med Indones 2010 42 123 129 20724765

[B44] Gupta A Brashear M Johnson W Prediabetes and prehypertension in healthy adults are associated with Low vitamin D levels Diabetes Care 2011 34 658 660 10.2337/dc10-1829 21282345 PMC3041202

[B45] Al-Daghri NM Alkharfy KM Al-Othman A El-Kholie E Moharram O Alokail MS Al-Saleh Y Sabico S Kumar S Chrousos GP Vitamin D supplementation as an adjuvant therapy for patients with T2DM: an 18-month prospective interventional study Cardiovasc Diabetol 2012 11 85 10.1186/1475-2840-11-85 22809461 PMC3461474

[B46] Elzubier AG AL-Shehry SZ Too many vitamins for diabetics World Health Forum 1997 18 73 74 9233078

[B47] Kienreich K Tomaschitz A Verheyen N Pieber T Gaksch M Grubler MR Pilz S Vitamin D and Cardiovascular Disease Nutrients 2013 5 3005 10.3390/nu5083005 23912328 PMC3775239

[B48] Abiaka C Delghandi M Kaur M Al-Saleh M Vitamin D status and anthropometric indices of an Omani study population Sultan Qaboos Univ Med J 2013 13 224 231 23862027 10.12816/0003227 PMC3706111

[B49] Al-Daghri NM Alkharfy KM Al-Othman A Yakout SM Al-Saleh Y Fouda M Sabico S Effect of non-pharmacologic vitamin D status correction on circulating bone markers in healthy overweight and obese Saudis Molecules 2013 18 10671 10680 10.3390/molecules180910671 24002141 PMC6269801

[B50] Dastani Z Berger C Langsetmo L Fu L Wong BY Malik S Goltzman D Cole DE Richards JB In healthy adults, biological activity of vitamin D, as assessed by serum PTH, is largely independent of DBP concentrations J Bone Miner Res 2013 [Epub ahead of print] 10.1002/jbmr.2042 23857798

[B51] Bentli R Taskapan H Toktas H Ulutas O Ozkahrahman A Comert M Significant predictors of vitamin D deficiency in inpatients and outpatients of a nephrology unit Int J Endocrinol 2013 2013 237869 23737771 10.1155/2013/237869 PMC3662121

[B52] Al-Saleh Y Al-Daghri NM Alkharfy KM Al-Attas OS Alokail MS Al-Othman A Sabico S Chrousos GP Normal circulating PTH in Saudi healthy individuals with hypovitaminosis D Horm Metab Res 2013 45 43 46 22972177 10.1055/s-0032-1323679

[B53] Al-Daghri NM Al-Attas OS Alokail MS Alkharfy KM El-Kholie E Yousef M Al-Othman A Al-Saleh Y Sabico S Kumar S Chrousos GP Increased vitamin D supplementation recommended during summer season in the gulf region: a counterintuitive seasonal effect in vitamin D levels in adult, overweight and obese Middle Eastern residents Clin Endocrinol (Oxf) 2012 76 346 350 10.1111/j.1365-2265.2011.04219.x 21906116

